# Sobrevida em Pacientes com Fenocópia de Brugada. Série de Casos

**DOI:** 10.36660/abc.20240526

**Published:** 2025-03-18

**Authors:** Luis Mariano De la Torre Fonseca, Robert Alarcón Cedeño, Lileska Andreína Hernandez Bello, Juan Díaz-Heredia, Diego Milton Pulla Quezada, Rubén Andrés Guamán Castro, Pablo Juan-Salvadores

**Affiliations:** 1 Hospital Comandante Manuel Fajardo Unit Care La Habana Cuba Hospital Comandante Manuel Fajardo – Unit Care, La Habana – Cuba; 2 Institute of Cardiovascular Health Isac-Med Interventional Cardiology Unit Guayaquil Equador Institute of Cardiovascular Health (Isac-Med) – Interventional Cardiology Unit, Guayaquil – Equador; 3 General Hospital of the North of Guayaquil Guayaquil Equador General Hospital of the North of Guayaquil, Guayaquil – Equador; 4 Universidad Catolica de Santiago de Guayaquil Guayaquil Equador Universidad Catolica de Santiago de Guayaquil – Cardriology, Guayaquil – Equador; 5 Institute of Cardiovascular Health Guayaquil Equador Institute of Cardiovascular Health – Cardiology, Guayaquil – Equador; 6 South Clinic Hospital Guayaquil Equador South Clinic Hospital – Emergency, Guayaquil – Equador; 7 University Hospital of Vigo Cardiology Department Vigo Espanha University Hospital of Vigo – Cardiology Department, Vigo – Espanha; 8 Galicia Sur Health Research Institute Vigo Espanha Galicia Sur Health Research Institute, Vigo – Espanha

**Keywords:** Síndrome de Brugada, Canais Iônicos, Relatos de Casos

## Introdução

A fenocópia de Brugada (FB) se refere a uma situação na qual os padrões eletrocardiográficos característicos da síndrome de Brugada (SB) são temporariamente manifestados, os quais são indistinguíveis dos padrões tipo 1 (coberto) e 2 (sela) da SB. Vários critérios adicionais devem ser considerados para um diagnóstico preciso. Estes incluem a presença de uma causa subjacente identificável, regressão do padrão uma vez que esta causa é corrigida, baixa probabilidade pré-teste de SB, um resultado negativo de um teste de indução farmacológica e um teste genético negativo.^1^

Atualmente, o verdadeiro mecanismo que causa a FB permanece desconhecido. Entretanto, a elevação do segmento ST poderia ser explicada pelo gradiente transmural resultante da perda da cúpula do potencial de ação no epicárdio e não no endocárdio ventricular. Esse fenômeno se origina do aumento transitório das correntes de saída de K (Ito) ou da diminuição das correntes de entrada de Ca tipo L e das correntes de pico de Na na fase 1 do potencial de ação. Várias condições clínicas reversíveis, como distúrbios do meio interno, compressão mecânica, isquemia e embolia pulmonar, doenças miocárdicas e pericárdicas, entre outras, constituem suas principais etiologias.^2^

Até o momento, o prognóstico da FB varia de acordo com a condição subjacente que a desencadeia e tende a ser mais favorável em comparação à SB. No entanto, estudos atuais avaliando a sobrevida de pacientes com essa condição são escassos. Este estudo teve como objetivo determinar a sobrevida de um grupo de pacientes hospitalizados em nosso centro com o diagnóstico de FB.

Nosso estudo incluiu 7 pacientes com padrão eletrocardiográfico de Brugada (Tipo 1), baixa probabilidade pré-teste para SB e condição clínica que poderia justificar a presença de sua fenocópia (material suplementar 1). Foram aplicados os critérios diagnósticos para FB descritos pelos principais especialistas da área (material suplementar 2); no entanto, devido às condições hemodinâmicas, o teste farmacológico com bloqueadores dos canais de sódio (Classe B) não foi possível. Uma vez resolvida a condição de base, a reversão do padrão eletrocardiográfico foi observada em todos os casos.

## Resultados

**Figura 1:**
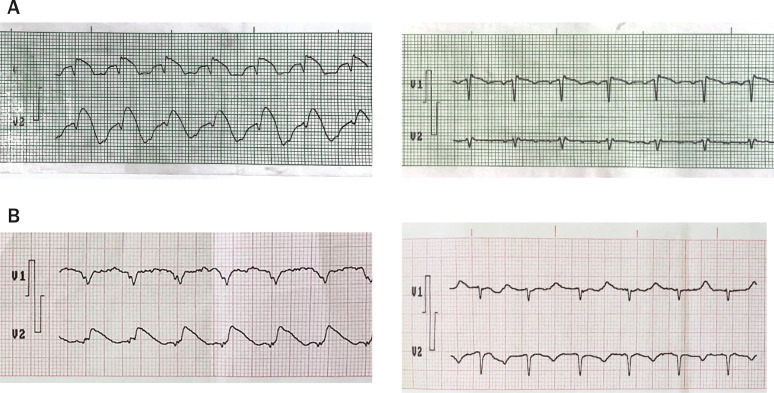
– Eletrocardiograma em dois pacientes com FB antes e depois do tratamento. A) Paciente com cetoacidose diabética e hipercalemia apresentando FB secundária a distúrbios eletrolíticos com resolução do padrão eletrocardiográfico após o tratamento. B) Paciente com infarto do miocárdio com supradesnivelamento do segmento ST apresentando FB, que se resolveu após terapia fibrinolítica.

Foram analisados os resultados de 7 pacientes com diagnóstico de FB internados em um hospital secundário durante o período de janeiro de 2018 a dezembro de 2023. O padrão predominante foi o tipo 1, e foi observada recuperação eletrocardiográfica completa após tratamento da causa de base (Figura 1). A média de idade foi de 70 anos ± 13,5, 57,1% eram do sexo feminino e o histórico pessoal mais frequente foi hipertensão arterial. Além disso, infarto do miocárdio com supradesnivelamento do segmento ST, choque séptico por COVID-19 e distúrbios de potássio (hipo e hipercalemia) ocorreram em 2 casos cada.

**Figura 2:**
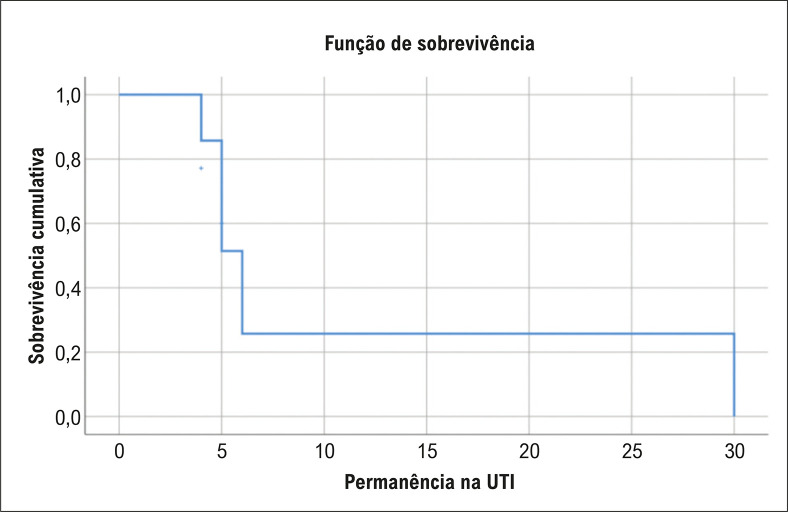
– Sobrevida global em pacientes com FB.

Em relação à ocorrência de arritmias cardíacas durante a internação, 3 pacientes apresentaram episódios de fibrilação atrial (42,9%), e apenas um paciente apresentou taquicardia ventricular. Em 2 pacientes, o padrão eletrocardiográfico da FB foi acompanhado de QTc maior que 470 ms. A mediana de internação hospitalar foi de 5 dias (ICR 4-6), e 5 pacientes (71,4%) morreram durante a admissão (Tabela 1). A sobrevida global intra-hospitalar foi de 26,8%, com seguimento mediano de 6 dias (IC 95%: 4,6 - 7,4) (Figura 2).

**Tabela 1:** – Características gerais

Variáveis	Caso 1	Caso 2	Caso 3	Caso 4	Caso 5	Caso 6	Caso 7
Idade	53	86	63	73	83	54	78
Sexo	Feminino	Feminino	Masculino	Feminino	Masculino	Feminino	Masculino
História pessoal	DM, obesidade, dislipidemia	Doença cardíaca isquêmica, HT	HT, DM	Não refere	HT	HT, obesidade	HT
Diagnóstico	Hipercalemia	IAMCSST	Embolia pulmonar	IAMCSST e Hipocalemia	Choque séptico (COVID-19)	Choque séptico (COVID-19)	IAMSST
QTc longo	Sim	Sim	Não	Não	Não	Sim	Não
Arritmias Ventriculares	Não	Sim	Não	Não	Não	Não	Não
Fibrilação atrial	Não	Sim	Sim	Não	Não	Não	Sim
Reversão de padrão (horas)	24	48	72	48	48	24	24
Permanência na unidade de terapia intensiva (dias)	5	6	30	5	4	5	4
Situação na alta	Vivo	Morto	Morto	Morto	Morto	Morto	Vivo
Temperatura (º C)	36	35,5	36	35	35,5	36	36,5
K (mmol/L)	7,47	5.10	4,80	1,80	4,80	3,80	5.04
Ca^2+^ (mmol/L)	0,97	0,82	1.12	0,95	0,96	1.06	0,73
Creatinina (mmol/L)	108	140	130	145	334	74	74

DM: diabetes mellitus; HT: hipertensão; IAMCSST: infarto do miocárdio com supradesnivelamento do segmento ST; IAMSST: infarto do miocárdio sem supradesnivelamento do segmento ST; UTI: unidade de terapia intensiva.

Atualmente, a maioria das evidências disponíveis neste campo se limita a relatos de casos e revisões sistemáticas. No entanto, as etiologias mais frequentes da FB coincidem com as encontradas nesta investigação: distúrbios do meio interno, isquemia miocárdica e embolia pulmonar, bem como infecção por COVID-19.^2-4^ As alterações causadas por essas doenças nos canais de saída de Ito e nos canais de entrada de Ca e Na na fase 1 do potencial de ação,^5^ bem como nos canais de K na fase 2, desencadeiam outras alterações elétricas, como prolongamento do intervalo QT, fibrilação atrial e taquicardia ventricular.

Nossa pesquisa representa uma das primeiras tentativas de avaliar a sobrevida em pacientes com FB. Os resultados do nosso estudo revelam uma taxa de sobrevida inferior a 30%, significativamente menor quando comparada aos resultados descritos por outros autores.^1,6^ A apresentação dessa entidade em pacientes com doenças graves com alta mortalidade, como choque séptico, infarto agudo do miocárdio e embolia pulmonar de alto risco, provavelmente influenciou esses resultados desfavoráveis. No entanto, a maioria dos pacientes relatados tinha doenças subjacentes graves; essas condições poderiam explicar a alta mortalidade relatada, impedindo uma reflexão precisa do prognóstico para todos os pacientes com FB.

Estudos multicêntricos com tamanhos de amostra maiores são necessários para entender melhor a FB. Embora a necessidade de mais investigação persista, certas etiologias, como distúrbios do meio interno, isquemia miocárdica e embolia pulmonar, são consistentes na maioria dos casos. Apesar da falta de consenso sobre o mecanismo exato da FB, parece evidente que essas condições contribuem significativamente para seu desenvolvimento. Além disso, nossas descobertas sugerem que a sobrevivência de pacientes com FB é relativamente inferior em comparação aos resultados de outras investigações. Essa discrepância destaca a importância de abordar de forma abrangente os fatores de risco e comorbidades associados à FB para melhorar os resultados clínicos e a qualidade de vida dos pacientes.
