# Supplementary material for: Sobrevida em Pacientes com Fenocópia de Brugada. Série de Casos
Source: Arq Bras Cardiol. 2025 Mar 18;122(3):e20240526. [Article in Portuguese] doi: 10.36660/abc.20240526 (PMC12013735; doi:10.36660/abc.20240526)

Supplementary material 1. Electrocardiogram (leads V1-V2) of patients with Brugada phenocopy.

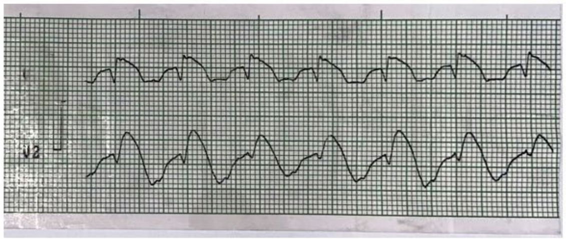

Case 1: Hyperkalemia

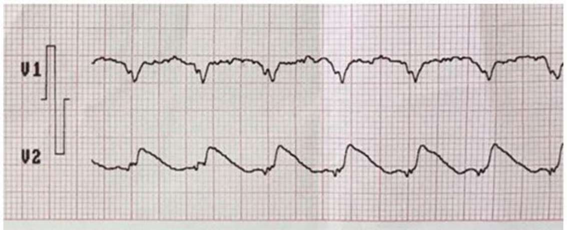

Case 2: STEMI and cardiogenic shock

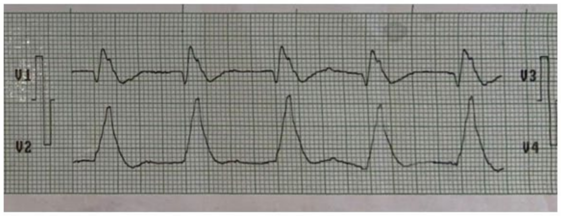

Case 3: Pulmonary embolism

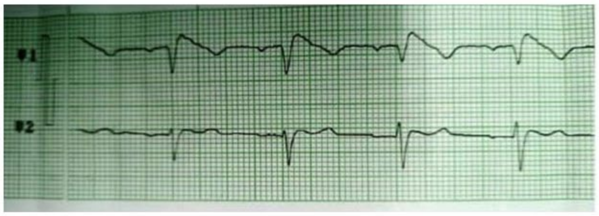

Case 2: STEMI

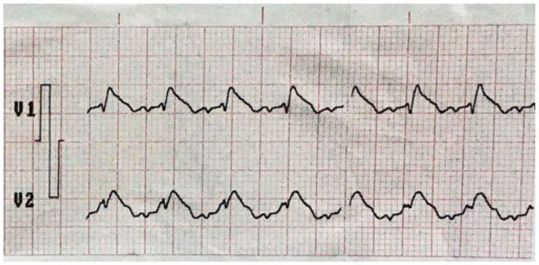

Case 5: COVID-19 and septic shock

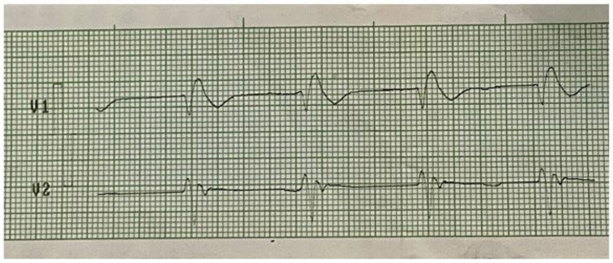

Case 6: COVID-19

Case 7:NSTEMI

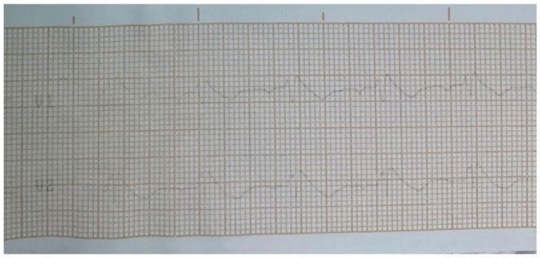

Supplement: Supplementary file 1 [file 0066-782X-abc-122-3-e20240526-suppl01.pdf]
