# Supplementary material for: Sobrevida em Pacientes com Fenocópia de Brugada. Série de Casos
Source: Arq Bras Cardiol. 2025 Mar 18;122(3):e20240526. [Article in Portuguese] doi: 10.36660/abc.20240526 (PMC12013735; doi:10.36660/abc.20240526)
Supplement: Supplementary file 2 [file 0066-782X-abc-122-3-e20240526-suppl02.pdf]

Supplementary material 2. Brugada Phenocopy Diagnostic Criteria

1. Presenting ECG pattern is of a type-1 or type-2 Brugada morphology identified by more than two expert cardiologists.

2. The patient presents with an identifiable underlying condition.

3. The ECG pattern normalizes after resolution of the underlying condition.

4. There is a low clinical pretest probability of true Brugada syndrome determined by lack of symptoms, medical history, and family history.

5. Provocation tests with sodium channel blocking agents such as ajmaline, flecainide or procainamide were not possible due to the hemodynamic condition of the patients, and were contraindicated in diseases such as acute coronary syndrome.

6. Genetic testing was not recommended due to its low reproducibility for Brugada syndrome (between 20% and 30%) and the hemodynamic condition of the patients.
