# Supplementary material for: Sobrevida em Pacientes com Fenocópia de Brugada. Série de Casos
Source: Arq Bras Cardiol. 2025 Mar 18;122(3):e20240526. [Article in Portuguese] doi: 10.36660/abc.20240526 (PMC12013735; doi:10.36660/abc.20240526)
Supplement: Translation

# Translation: Survival in Patients with Brugada Phenocopy. Case Series

**DOI:** 10.36660/abc.20240526i

**Authors:** Luis Mariano De la Torre Fonseca, Robert Alarcón Cedeño, Lileska Andreína Hernandez Bello, Juan Díaz-Heredia, Diego Milton Pulla Quezada, Rubén Andrés Guamán Castro, Pablo Juan-Salvadores

**Affiliations:** 1 Cuba Hospital Comandante Manuel Fajardo – Unit Care, La Habana – Cuba; 2 Ecuador Institute of Cardiovascular Health (Isac-Med) – Interventional Cardiology Unit, Guayaquil – Ecuador; 3 Ecuador General Hospital of the North of Guayaquil, Guayaquil – Ecuador; 4 Ecuador Universidad Catolica de Santiago de Guayaquil – Cardriology, Guayaquil – Ecuador; 5 Ecuador Institute of Cardiovascular Health – Cardiology, Guayaquil – Ecuador; 6 Ecuador South Clinic Hospital – Emergency, Guayaquil – Ecuador; 7 Spain University Hospital of Vigo – Cardiology Department, Vigo – Spain; 8 Spain Galicia Sur Health Research Institute, Vigo – Spain

**Keywords:** Brugada Syndrome, Ion Channels, Case Reports

## Introduction

Brugada phenocopy (BP) refers to a situation in which the characteristic electrocardiographic patterns of Brugada syndrome (BS) are temporarily manifested, which are indistinguishable from the type 1 (coved) and 2 (saddleback) patterns of BS. Several additional criteria must be considered for an accurate diagnosis. These include the presence of an identifiable underlying cause, regression of the pattern once this cause is corrected, low pretest probability of BS, a negative result of a pharmacological induction test, and a negative genetic test.^1^

Currently, the true mechanism that causes BP remains unknown. However, ST-segment elevation could be explained by the transmural gradient resulting from the loss of the action potential dome in the epicardium and not in the ventricular endocardium. This phenomenon originates from the transient increase in K outward currents (Ito) or the decrease in L-type Ca inward currents and peak Na currents in phase 1 of the action potential. Various reversible clinical conditions, such as disorders of the internal environment, mechanical compression, ischemia and pulmonary embolism, myocardial and pericardial diseases, among others, constitute its main etiologies.^2^

So far, the prognosis of BP varies according to the underlying condition that triggers it and tends to be more favorable compared to BS. However, current studies evaluating the survival of patients with this condition are scarce. This study aimed to determine the survival of a group of patients hospitalized in our center with the diagnosis of BP.

Our study included 7 patients with the electrocardiographic pattern of Brugada (Type 1), a low pretest probability for BS, and a clinical condition that could justify the presence of its phenocopy. (supplementary material 1) The diagnostic criteria for BP described by leading experts in the field were applied (supplementary material 2); however, due to hemodynamic conditions, the pharmacological test with sodium channel blockers (Class B) was not possible. Once the underlying condition was resolved, the reversal of the electrocardiographic pattern was observed in all cases.

## Results

The results of 7 patients with a diagnosis of BP hospitalized in a secondary hospital during the period from January 2018 to December 2023 were analyzed. The predominant pattern was type 1, and complete electrocardiographic recovery was observed following treatment of the underlying cause (Figure 1). The mean age was 70 years ± 13.5, 57.1% were female, and the most frequent personal history was arterial hypertension. Additionally, ST-segment elevation myocardial infarction, COVID-19 septic shock, and potassium disorders (hypo- and hyperkalemia) occurred in 2 cases each.

**Figure 1:** – Electrocardiogram in two patients with BP before and after treatment. A) Patient with diabetic ketoacidosis and hyperkalemia presenting with a BP secondary to electrolyte disturbances with resolution of the electrocardiographic pattern following treatment. B) Patient with ST-segment elevation myocardial infarction presenting with a BP, which resolved after fibrinolytic therapy.

Regarding the occurrence of cardiac arrhythmias during hospitalization, 3 patients presented episodes of atrial fibrillation (42.9%), and only one patient exhibited ventricular tachycardia. In 2 patients, the electrocardiographic pattern of BP was accompanied by a QTc greater than 470 ms. The median hospital stay was 5 days (ICR 4-6), and 5 patients (71.4%) died during admission (Table 1). Overall in-hospital survival was 26.8%, with a median follow-up of 6 days (95% CI: 4.6 - 7.4) (Figure 2).

**Table 1:** – General characteristics

Variables	Case 1	Case 2	Case 3	Case 4	Case 5	Case 6	Case 7
Age	53	86	63	73	83	54	78
Sex	Female	Female	Male	Female	Male	Female	Male
Personal history	DM, obesity, dyslipidemia	Ischemic heart disease, HT	HT, DM	No refer	HT	HT, obesity	HT
Diagnosis	Hyperkalemia	STEMI	Pulmonary embolism	STEMI and Hypokalemia	Septic shock (COVID-19)	Septic shock (COVID-19)	NSTEMI
Long QTc	Yes	Yes	No	No	No	Yes	No
Ventricular Arrhythmias	No	Yes	No	No	No	No	No
Atrial Fibrillation	No	Yes	Yes	No	No	No	Yes
Pattern reversal (hours)	24	48	72	48	48	24	24
Intensive Care unit stay (days)	5	6	30	5	4	5	4
Status at discharge	Alive	Deceased	Deceased	Deceased	Deceased	Deceased	Alive
Temperatur (º C)	36	35.5	36	35	35.5	36	36.5
K (mmol/L)	7.47	5.10	4.80	1.80	4.80	3.80	5.04
Ca (mmol/L)	0.97	0.82	1.12	0.95	0.96	1.06	0.73
Creatinine (mmol/L)	108	140	130	145	334	74	74

DM: diabetes mellitus; HT: hypertension; STEMI: ST-segment elevation myocardial infarction; NSTEMI: Non-ST-segment elevation myocardial infarction; ICU: intensive care unit.

**Figure 2:** – Overall survival in patients with BP.

At present, most of the available evidence in this field is limited to case reports and systematic reviews. Nevertheless, the most frequent etiologies of BP coincide with those found in this investigation: internal milieu disorders, myocardial ischemia, and pulmonary embolism, as well as COVID-19 infection.^2-4^ The alterations caused by these diseases on Ito outflow channels and Ca and Na inflow channels in phase 1 of the action potential,^5^ as well as K channels in phase 2, trigger other electrical alterations such as prolongation of the QT interval, atrial fibrillation, and ventricular tachycardia.

Our research represents one of the first attempts to evaluate survival in BP patients. The results of our study reveal a survival rate of less than 30%, significantly lower when compared to the results described by other authors.^1,6^ The presentation of this entity in patients with severe diseases with high mortality, such as septic shock, acute myocardial infarction, and high-risk pulmonary embolism, probably influenced these unfavorable results. However, most of the reported patients had serious underlying diseases; these conditions could explain the high mortality reported, preventing an accurate reflection of the prognosis for all patients with BP.

Multicenter studies with larger sample sizes are needed to understand BP better. Although the need for further investigation persists, certain etiologies, such as internal milieu disorders, myocardial ischemia, and pulmonary embolism, are consistent in most cases. Despite the lack of consensus on the exact mechanism of BP, it seems evident that these conditions contribute significantly to its development. Furthermore, our findings suggest that the survival of patients with BP is relatively inferior compared to the results of other investigations. This discrepancy highlights the importance of comprehensively addressing the risk factors and comorbidities associated with BP to improve clinical outcomes and quality of life for patients.
